# Extracorporeal membrane oxygenation in long-term COVID-19 with severe neutropenia and thrombocytopenia after allogeneic hematopoietic stem cell transplantation: a case report

**DOI:** 10.1186/s12879-024-09121-6

**Published:** 2024-02-20

**Authors:** Shiqi Guo, Linna Zhang, Chang Gao, Xiaoting Lu, Wei Song, Hui Shen, Qiang Guo

**Affiliations:** 1 Department of Pulmonary and Critical Care Medicine, The Fourth Affiliated Hospital of Soochow University (Suzhou Dushu Lake Hospital), Suzhou, Jiangsu China; 2 Department of Emergency, the Fourth Affiliated Hospital of Soochow University (Suzhou Dushu Lake Hospital), Suzhou, Jiangsu China; 3 Department of Emergency, The Affiliated Hospital of Xuzhou Medical University, Xuzhou, Jiangsu China; 4 Department of Emergency and Critical Care Medicine, The First Affiliated Hospital of Soochow University, Suzhou, Jiangsu China

**Keywords:** Acute respiratory distress syndrome (ARDS), Extracorporeal membrane oxygenation (ECMO), Hematopoietic stem cell transplantation (HSCT), COVID-19

## Abstract

**Background:**

Hematopoietic stem cell transplantation (HSCT) was associated with potentially life-threatening complications. Among patients supported by extracorporeal membrane oxygenation (ECMO), those who underwent HSCT had a worse prognosis than those who did not. Advances in HSCT and critical care management have improved the prognosis of ECMO-supported HSCT patients.

**Case:**

The patient in the remission stage of lymphoma after 22 months of allogeneic hematopoietic stem cell transplantation, suffered from ARDS, severe neutropenia, thrombocytopenia, and long-term COVID-19. We evaluated the benefits and risks of ECMO for the patient, including the possibility of being free from ECMO, the status of malignancy, the interval from HSCT to ARDS, the function of the graft, the amount of organ failure, and the comorbidities. ECMO was ultimately used to save his life.

**Conclusions:**

We did not advocate for the general use of ECMO in HSCT patients and we believed that highly selected patients, with well-controlled tumors, few comorbidities, and fewer risk factors for death, tended to benefit from ECMO with well ICU management.

## Background

Hematopoietic stem cell transplantation (HSCT) was associated with potentially life-threatening complications such as opportunistic infections, graft-versus-host disease (GVHD), and relapse of the underlying disease. The development of acute respiratory distress syndrome (ARDS) is also a huge hit for these patients. The complexity of HSCT and the critical presentation of ARDS made it difficult for these patients to benefit from ECMO therapy [1, 2]. The in-hospital mortality for HSCT patients using ECMO was significantly higher than those who did not receive HSCT [3–5]. ECMO is a highly technical life-saving intervention, and given the resources required and the practical benefits gained, ECMO was rarely used in patients with HSCT.

However, with post-transplant immune reconstitution and the gradual dose reduction of immunosuppressive drugs [6], the prognosis of patients supported with ECMO in late period of HSCT were better than those in early period of HSCT [4, 7]. The improving outcomes observed in the study suggested that highly selected HSCT patients may benefit from ECMO. Recently, an expert consensus on ECMO therapy in patients with HSCT stated that HSCT was not an absolute contraindication to ECMO and give suggestions for refining ECMO selection criteria and management in patients receiving HSCT [8]. Based on this, we successfully treated an allo-HSCT patient with ARDS on ECMO.

We searched PubMed with the following terms ‘HSCT + ECMO’ but did not find relevant research or report after the expert consensus. Thus, we decided to report this case to give reference for clinical practice and call for systematic research.

## Case presentation

**Fig. 1:**
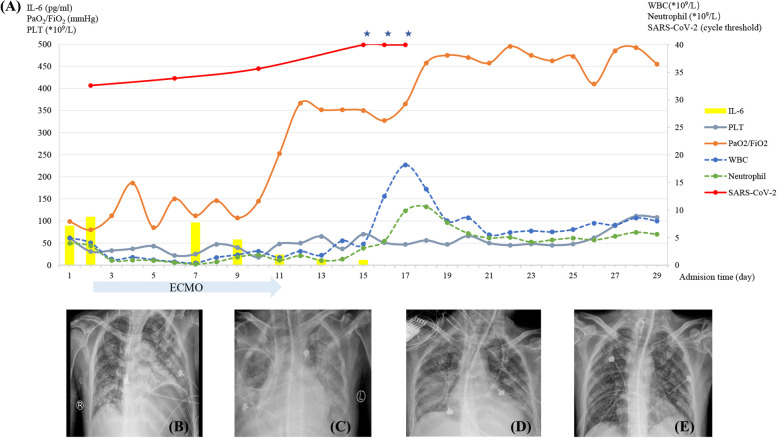
The clinical course of this patient. **A** Dynamic presentation of IL-6, PLT, PaO_2_/FiO_2_, WBC, Neutrophil, and the cycle threshold of SARS-CoV-2. From day 2 to day 11, the patient was experiencing ECMO support. * From day 15 to day 18, the cycle thresholds of Sars-CoV-2 were more than 40, which means, the tests for SARS-CoV-2 were negative. **B** On day 1, X-ray chest radiographs of the patient on admission to the EICU. **C** On day 2, before the use of V-V ECMO. **D** On day 11, free from ECMO. **E** On day 29, ventilator withdrawal

A 38-year-old man was transferred to this hospital because of severe cough and breathlessness worsened progressively for one week. On December 16, 2022, it was confirmed that he infected with Severe Acute Respiratory Syndrome Coronavirus-2 (SARS-CoV-2) for the first time. Although treatments with Nematovir/ritonavir (3 weeks totally), Azovudine (2 weeks totally), and Molnupiravir (4 weeks totally) were administered, the nucleic acid test for SARS-CoV-2 remained positive consistently. The patient suffered from lymphoma for 5 years, and underwent allogeneic hematopoietic stem cell transplantation (allo-HSCT) 22 months ago, achieving complete remission and took tacrolimus for 16 months as well as methylprednisolone for 6 months after allo-HSCT. On admission, this patient was received oxygen through a non-invasive ventilation (NIV), with positive end-expiratory pressure (PEEP) of 8 cm of water and fraction of inspired oxygen (FiO_2_) of 0.5. His temperature was 38.5℃, with the blood pressure of 85/50 mmHg, the heart rate of 110 beats/minute, and the oxygen saturation of 89%. Both lungs were heard pronounced wet rales. Arterial blood gas (ABG) analysis showed PaO_2_ at 55 mmHg, with a PaO_2_/FiO_2_ ratio (PFR) of 110 mmHg and lactate levels at 2.16 mmol/L; NT-proBNP and procalcitonin (PCT) levels were 72 pg/ml (< 125 pg/ml) and 4.65 ng/ml (< 0.046 ng/ml), respectively. In conjunction with the chest radiographs (Fig. 1B) at the time of admission, diagnosis included ARDS and long-term COVID-19. For no improving of the dyspnea and hypoxia within a span of two hours NIV, invasive mechanical ventilation (IMV) was initiated. Subsequent results from next-generation sequencing (NGS) on bronchoalveolar lavage indicated the presence of SARS-CoV-2. We did not use wonder anti-SARS-CoV-2 drugs, but methylprednisolone (80 mg for 3 days followed with 40 mg for 2 days) was used to suppress the excessive inflammatory response, and gamma globulin (20 g for 5 days) was used to assist in anti-infection and immunomodulation. Despite giving prone positioning and applying high PEEP of 15 cm H_2_O, PFR remained below 80 mmHg more than 6 h. In subsequent chest X-rays, there was a noticeable progression in the extent of pneumonia compared to admission (Fig. 1C). Catheters (right femoral vein: 21F, right internal jugular vein: 17F) for veno-venous extracorporeal membrane oxygenation (V-V ECMO) were inserted. Unfractionated heparin was used for anticoagulation, maintaining activated partial thromboplastin time (APTT) at 50–60 s. on day 2. The ECMO settings included a blood flow rate of 4.5 L/min and an oxygen flow rate of 5 L/min. This was accompanied by continued invasive ventilation using the volume-controlled mode with a PEEP of 10 cm H_2_O, FiO_2_ 0.6, a tidal volume of 280 ml, and a respiratory rate of 10 breaths/minute. The patient was also placed in the prone position for 16 h daily. Human Granulocyte Colony-stimulating Factor Injection (300ug, once a day, from day 3 to day 17), Recombinant Human Granulocyte/Macrophage Colony-stimulating Factor for Injection(300ug, once a day, from day 7 to day 17), Recombinant Human Thrombopoietin Injection (15000ug, once a day, from day 17 to day 27) and Herombopag Olamine Tablets (10 mg, once a day, from day 10 to day 28) were used due to the onset of neutropenia (with a nadir of 0.19 × 10^9^/L on day 6) and thrombocytopenia (with a minimum value of 22 × 10^9^/L on day 7). A total of 12 standard therapeutic doses of platelets, 4875 ml of plasma and 23.5 units of concentrated red blood cells were transfused during the hospitalization. On day 11, after short suspension of ECMO support and ramping up the ventilator support, PFR remained more than 250 mmHg for 2 h, ECMO was weaned off successfully. SARS-CoV-2 was negative after admission 15 days, and granulocytopenia and thrombocytopenia gradually recovered on day 11 and day 16 respectively. On day 29, he was free from IMV and transitioned to high-flow nasal cannula oxygen therapy and discharged on day 45.

## Discussion and conclusions

In this case, the patient in the remission stage of lymphoma after 22 months of transplantation, suffered from ARDS, severe neutropenia, thrombocytopenia, and long-term COVID-19. Kochanek and Hermann et al. concluded that platelet count is a major determinant of adverse prognosis on ECMO [9, 10]. DiNardo et al. also suggested in an expert consensus that ECMO should not be used in patients with platelets < 20*10^9^/L [8]. In the case we reported, the patient had thrombocytopenia and the platelet count prior to ECMO initiation was 31*10^9^/L. After communication with the patient's family, the decision was finally made to initiate ECMO, with platelet transfusion. In addition, for this thrombocytopenic patient, unfractionated heparin was used for anticoagulation. We tested his APTT to ensure that it was maintained at 50–60 s. This patient had no thrombotic or bleeding events during treatment.

Both ICU admissions and ECMO initiation increased during the COVID-19 epidemic [11, 12]. The experience with ECMO during the COVID-19 period remained valuable, including the timing of ECMO initiation and cessation, prognostic risk factors, and increased public awareness of ECMO. Furthermore, ECMO is not contraindicated in HSCT patients with COVID-19-induced ARDS. These experiences made potentially life-saving interventions available to HSCT patients, provided a reference for expected outcomes in HSCT patients, and guided decision making about ECMO.

DiNardo et al. summarized the clinical characteristics of HSCT patients must be concerned before receiving ECMO in an expert consensus, including the possibility of been free from ECMO, the status of malignancy, type of HSCT, the interval from HSCT to ARDS, GVDH prophylaxis and treatment, the function of the graft, the amount of organ failure, and the comorbidities [8]. Barbaro et al. also emphasized that mortality in ECMO patients was related to patient selection and patient treatment [12]. We did not recommend the general use of ECMO in HSCT patients. We believed that only highly selected patients, with well-controlled tumors, few comorbidities, and fewer risk factors for death, tended to benefit from ECMO with rigorous evaluation of indications and proper ICU management.

## Data Availability

All data generated or analyzed during this study are included in this published article and its supplementary information files.

## Abbreviations

ARDS: Acute respiratory distress syndrome
APTT: Activated partial thromboplastin time
Allo-HSCT: Allogeneic hematopoietic stem cell transplantation
ABG: Arterial blood gas
ECMO: Extracorporeal membrane oxygenation
HSCT: Hematopoietic stem cell transplantation
IMV: Invasive mechanical ventilation
NIV: Non-invasive ventilation
NGS: Next-generation sequencing
PCT: Procalcitonin
PFR: PaO_2_/FiO_2_ ratio
PEEP: Positive end-expiratory pressure
V-V ECMO: Veno-venous extracorporeal membrane oxygenation
WBC: White blood cells
